# Supplementary material for: A Humanized Yeast Phenomic Model of Deoxycytidine Kinase to Predict Genetic Buffering of Nucleoside Analog Cytotoxicity
Source: Genes (Basel). 2019 Sep 30;10(10):770. doi: 10.3390/genes10100770 (PMC6826991; doi:10.3390/genes10100770)
Supplement: Supplementary file 1 [file genes-10-00770-s001.zip › Additional_Files/Additional_File7_GOTermHeatmaps.zip!/Additional_File7_GOTermHeatmaps/90S_preribosome.pdf]

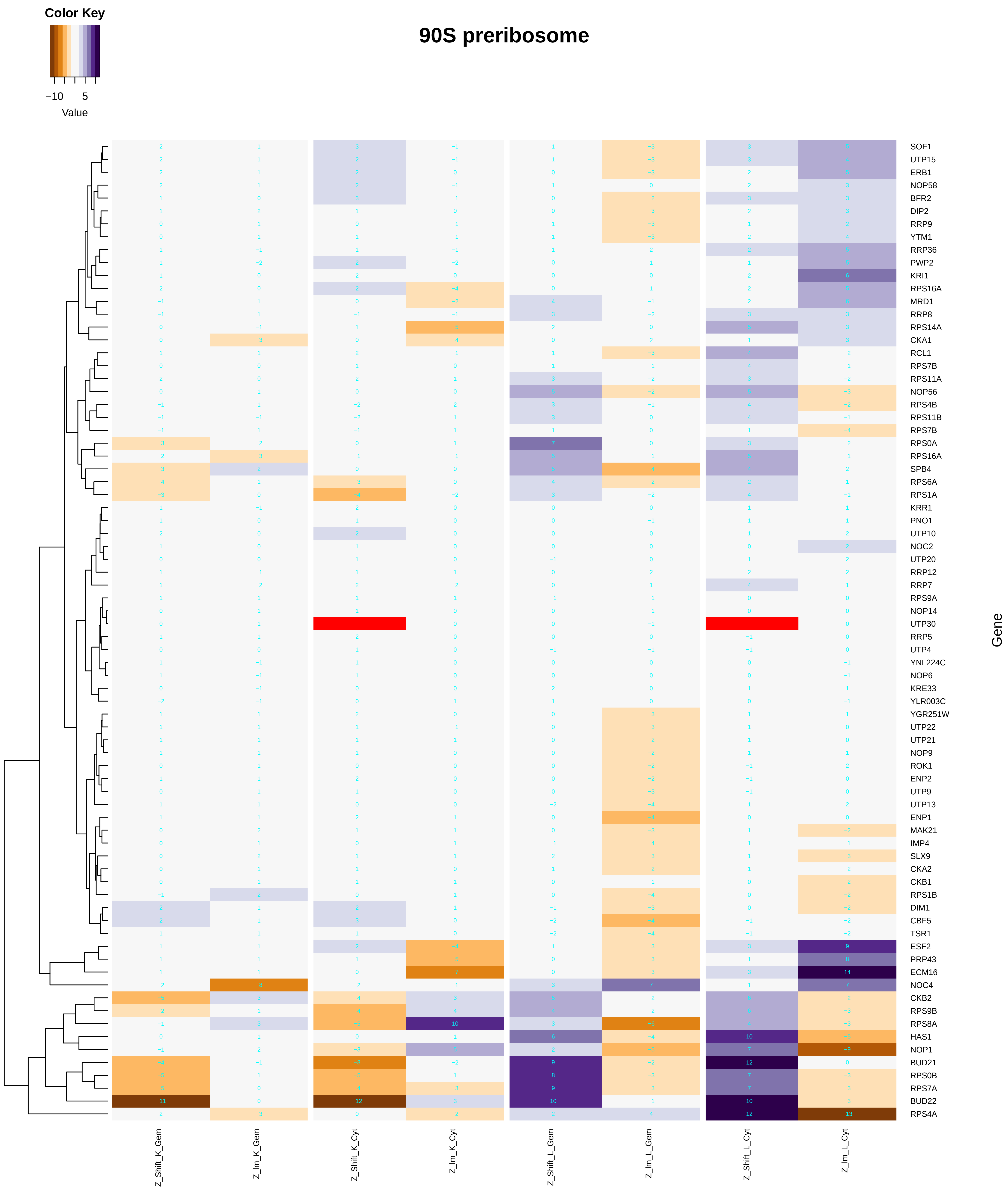

90S preribosome
Color Key
−10
5
Value
Gene
SOF1
UTP15
ERB1
NOP58
BFR2
DIP2
RRP9
YTM1
RRP36
PWP2
KRI1
RPS16A
MRD1
RRP8
RPS14A
CKA1
RCL1
RPS7B
RPS11A
NOP56
RPS4B
RPS11B
RPS7B
RPS0A
RPS16A
SPB4
RPS6A
RPS1A
KRR1
PNO1
UTP10
NOC2
UTP20
RRP12
RRP7
RPS9A
NOP14
UTP30
RRP5
UTP4
YNL224C
NOP6
KRE33
YLR003C
YGR251W
UTP22
UTP21
NOP9
ROK1
ENP2
UTP9
UTP13
ENP1
MAK21
IMP4
SLX9
CKA2
CKB1
RPS1B
DIM1
CBF5
TSR1
ESF2
PRP43
ECM16
NOC4
CKB2
RPS9B
RPS8A
HAS1
NOP1
BUD21
RPS0B
RPS7A
BUD22
RPS4A
Z_Shift_K_Gem
Z_Im_K_Gem
Z_Shift_K_Cyt
Z_Im_K_Cyt
Z_Shift_L_Gem
Z_Im_L_Gem
Z_Shift_L_Cyt
Z_Im_L_Cyt
